# Parametric and Nonparametric Analysis of the Internal Structure of the Psychosocial Work Processes Questionnaire (PROPSIT) as Applied to Workers

**DOI:** 10.3390/ijerph19137970

**Published:** 2022-06-29

**Authors:** César Merino-Soto, Arturo Juárez-García, Guillermo Salinas Escudero, Filiberto Toledano-Toledano

**Affiliations:** 1 Instituto de Investigación de Psicología, Universidad de San Martín de Porres, Lima 15102, Peru; sikayax@yahoo.com.ar; 2 Centro de Investigación Transdisciplinar en Psicología, Universidad Autónoma del Estado de Morelos, Pico de Orizaba 1, Los Volcanes, Cuernavaca 62350, Mexico; arturojuarezg@hotmail.com; 3 Centro de Estudios Económicos y Sociales en Salud, Hospital Infantil de Mexico Federico Gómez, National Institute of Health, Márquez 162, Doctores, Cuauhtémoc, Mexico City 06720, Mexico; guillermosalinas@yahoo.com; 4 Unidad de Investigación en Medicina Basada en Evidencias, Hospital Infantil de Mexico Federico Gómez, National Institute of Health, Márquez 162, Doctores, Cuauhtémoc, Mexico City 06720, Mexico; 5 Unidad de Investigación Sociomédica, Instituto Nacional de Rehabilitación Luis Guillermo Ibarra, Calzada México-Xochimilco 289, Arenal de Guadalupe, Tlalpan, Mexico City 14389, Mexico

**Keywords:** psychosocial risk factors, labor resources, work stress, factor analysis

## Abstract

The study of the dimensionality or internal structure of a measure has a definitional purpose with notable theoretical and practical implications; this aspect can be analyzed via both parametric and nonparametric approaches. The latter are probably used less often to validate constructs in the context of psychosocial work factors. The aim of the present manuscript was to employ both nonparametric (DETECT and AISP-Mokken) and parametric (semiconfirmatory factor analysis) procedures to analyze the internal structure of the Psychosocial Work Processes Questionnaire (PROPSIT) in the context of two samples of Peruvian workers located in the city of Lima, Perú, with one sample drawn from various work centers (n = 201) and the other comprising elementary education teachers (n = 158). The nonparametric results indicated that the content of the PROPSIT is sufficiently multidimensional to be able to describe a variety of psychosocial factors, while the parametric results require modification of the measurement model to obtain greater factorial congruence. In general, the analyses show a similar structure to those discussed by previous preliminary studies that have reported similar item-level performances. Some findings and considerations for future research are discussed.

## 1. Introduction

### 1.1. Parametric and Nonparametric Analysis of Dimensionality

The study of the dimensionality or internal structure of a measure has a definitional purpose for both theory and practice in terms of the use of any psychosocial instrument. However, certain conceptual differences, such as the difference between classical or strict dimensionality and essential dimensionality, which has been recognized for some years and from which guidelines for obtaining valid conclusions regarding such an internal structure have been derived, must be considered in dimensionality assessment procedures. Classical dimensionality involves the identification of totally homogeneous, unidimensional structures exhibiting local independence, whereas essential dimensionality focuses on a common predominant factor alongside additional factors shared among subsets of components, which seems to be more closely related to the theoretical needs and purposes of psychological instruments [1,2,3]. This differentiation is also important to deepen not only our ability to theorize about a given construct but also our capacity to apply the assumptions that are inherent to the mathematical procedures used to assess it.

Similar to other statistical procedures, dimensionality research can be analyzed via parametric and nonparametric approaches, and the latter are probably used less often in the context of construct validation with respect to psychosocial work factors. The nonparametric approach has proven to be efficient and accurate in identifying the essential dimensionality of a construct [4], even under conditions in which a relatively small sample size presents typical challenges [5]. These advantages also include the approach being effective for processing scales with few items [5,6,7]. One such methodology, which is based on conditional covariance between items, is the DETECT algorithm (the Dimensionality Evaluation to Enumerate Contributing Traits index; [8]), which has been shown by simulation studies and empirical data to correctly identify dimensionality [4,9].

However, although nonparametric approaches are efficient when applied to moderately small samples, the results of other validation strategies must be considered carefully to prevent false positives in identifying dimensionality. For example, cross-validation strategies applied to the sample itself [10] and the implementation of parametric procedures [11] are additional tools that can serve as corroborating evidence in validating a measure.

The study of dimensionality considers, among other factors, statistical assumptions pertaining to the data and sample size [12]; for example, when conducting a parametric assessment of dimensionality by structural equation modeling (SEM), large samples are expected, particularly in the context of multidimensional measures. However, in the context of a multidimensional measure, one can also look for unidimensional structures [13,14]; that is, the assessment can be performed differentially for each type of content in the context of the overall contents. This approach alleviates the difficulties that occur when analyzing small samples pertaining to measures containing a small number of items, and it is also a reasonable approximation of dimensionality that does not sacrifice precision.

### 1.2. The Psychosocial Work Processes Questionnaire (PROPSIT)

The Joint Committee of the International Labor Organization and the World Health Organization [15] has recognized that psychosocial processes at work are complex and difficult to understand, since they include factors pertaining to organizational context and the task at hand, the individual psychological dispositions of the worker, extra occupational aspects, psychological effects and general health indicators. Although all these dimensions are important, the literature has particularly focused on factors related to organizational context and the characteristics of the task, which are understood as demands or psychosocial labor factors to which the worker is exposed and are known to influence the health–illness process through the biopsychosocial mechanisms of stress [16]. There are few instruments available to measure such factors, particularly in a Latin American context, which makes it necessary to develop ways of measuring these complex phenomena.

The aim of the present study was to improve the effectiveness of evaluating dimensionality via the questionnaire for the evaluation of psychosocial processes at work (PROPSIT; [17]), a new measure created to identify and evaluate work-related psychosocial factors, active moderators, effects on health and well-being, and other intervening psychosocial variables in Mexican workers. The theoretical models forming the framework for this measure were the demand–control model [18], the effort–reward model [19], and in particular, the job demands–resources model [20,21,22]. As in the case of those models, in the PROPSIT model, both negative factors (of psychosocial risk) and positive or salutogenic factors interact with each other to define the motivational and health–illness processes of the worker [17]. Likewise, in the PROPSIT model, the negative impacts of psychosocial risk factors (PRFs, e.g., psychological demands or harassment at work) are also considered. For example, psychological demands, workplace harassment, and strict supervision are moderated by the presence of salutogenic factors or resources (control over work, social support, job resources, etc.). If such salutogenic resources are encountered more frequently than risk factors, the probability of favorable psychological effects such as engagement and motivation, in general, increases, while if encounters with risk factors exceed resources, negative stress, burnout and illness ensue [16].

In addition to the influence of the aforementioned models, the PROPSIT was developed based on previous qualitative studies [23,24,25], which revealed the need for an exclusive instrument for use in the Latin American context, given the cultural differences and semantic limitations of other instruments created in industrialized countries [26]. Using a new qualitative–quantitative method in several iterations [23,24,25], work events that were perceived by workers as work stressors or sources of engagement were explored. These perceptions were categorized and structured into various dimensions and verified in other Mexican worker samples [26]. Furthermore, although the PROPSIT was constructed to represent labor variables relevant to Mexican workers with a formal employment contract, it is potentially generalizable to other Latin American contexts because its dimensions also represent etic constructs that have been integrated into other models and consistently identified in many international studies [18,19,27]. The generalization of these constructs, however, is not only a matter of assumptions but also of empirical evidence, so contrast with data from the new context of instrument use is needed.

In the initial version of the PROPSIT, eight dimensions were proposed to assess psychosocial factors: three risk factors (job demands, harassment, and stressful leadership) and five salutogenic resources (rewards, control over work, material resources for work, climate of social support and congruence of values). However, in a first validation in the context of blue-collar workers in Mexico, in terms of risk factors, job demands were divided into physical and psychological demands, and psychological harassment was combined with stressful leadership for a total of three dimensions. In terms of favorable resources, three dimensions were ultimately used: supportive climate and values were combined into one dimension, as were rewards and resources, with only the dimension of control over work remaining independent [17]. However, there is a clear need for further studies to be conducted to determine the appropriate number and grouping of factors.

This instrument is potentially useful for the Peruvian context, given the still limited development of occupational psychology in Peru, which has also resulted in a limited range of measures for psychosocial factors. To date, the development of theoretical or applied scientific research in Peru on this topic is only emerging in relation to both psychometric and nonpsychometric studies [28,29]. The existing validations e.g., [30,31,32] cover only a narrow range of validity evidence because (1) they were mainly limited to assessing internal structure, specifically dimensionality; (2) they obtained estimates of only one type of reliability (internal consistency); (3) they were developed mostly with workers in Metropolitan Lima; (4) their decisions on item changes were mainly empirical, without theoretical justification or support from expert reviewers; (5) it is unknown whether response tendencies irrelevant to the construct, especially referring to social desirability, were documented and controlled in the studies; (6) some equivalence of psychometric properties between groups was not verified; and (7) the interpretation of the results inappropriately generalized scope of the specific validity evidence investigated to other types of evidence, a situation known as validity induction [33].

### 1.3. Objective and Hypothesis

Given the factors mentioned above and the need for more studies of the PROPSIT in different occupational and geographical contexts, the purpose of this manuscript was to analyze the dimensional structure of the PROPSIT using both parametric and nonparametric strategies to examine a sample of Peruvian workers drawn from different labor sectors. Hypotheses were established regarding the dimensionality of the PROPSIT as follows:

**Hypothesis** **1** **(H1).** *The PROPSIT exhibits a multidimensional structure with oblique dimensions. According to the theoretical model and empirical results of the examination of psychosocial work factors by Juárez-García and Flores-Jiménez [17], there are several nonredundant, independent and associated dimensions that characterize psychosocial risk factors (PRFs) and favorable resources. Therefore, the items are expected to represent unidimensionality with respect to their own scoring*.

**Hypothesis** **2** **(H2).** *These dimensions are statistically distinct. Although the latent psychosocial dimensions are realistically expected to maintain some degree of coverability, the degree to which this association varies is a consequence of measurement error and substantive theory, among other factors [12]. According to a study by Juárez-García and Flores-Jiménez [17], the latent dimensions in the PRF area (psychological demands, physical demands, and psychological harassment) covaried at r_mean_ = 0.329 and exhibited apparently homogeneous correlations. This result indicates that the dimensions can be expressed as moderately independent latent variables. On the other hand, in accordance with a theoretically guided construction [17], the dimensions should not be highly related*.

The degree to which this objective was fulfilled was evaluated with two approaches: nonparametric and parametric. The first approach was exploratory and intended to produce a first approximation of dimensionality with respect to a relatively small sample and a small number of items pertaining to the specific facets of each PROPSIT dimension. The other approach, which was parametric, was applied to a larger sample size and provided the parameters that are usually estimated to define the internal structure of a measure during the factor analysis procedure.

## 2. Materials and Methods

### 2.1. Participants

#### 2.1.1. Nonparametric Approach

A total of 201 participants were sampled nonprobabilistically with the intention of ensuring heterogeneity in terms of their jobs and careers to maximize the number of work stressors to which they were exposed based on their work contexts, thereby potentially ensuring the robustness of the PROPSIT dimensions. The eligibility criteria were having reached the age of maturity, being Peruvian, being a worker with a formal contract, being currently employed, and giving voluntary assent to participate. Descriptive information on the participants can be seen in Table 1.

#### 2.1.2. Parametric Approach

The samples of participants included in this analysis were twofold: a group of workers with various positions and jobs (n = 201) and regular elementary education teachers from various public institutions located in Lima (n = 158). Descriptive information on this subject can also be seen in Table 1.

### 2.2. Measures

#### 2.2.1. The Psychosocial Work Processes Questionnaire (PROPSIT)

This questionnaire is a measurement of relevant work factors, effects and processes intended to evaluate psychosocial work conditions and their effects on the worker’s well-being. It contains four general sections: psychosocial factors at work (PFW), psychosocial effects of work, health-disease process and other extra-work psychosocial factors. The present study validated the PFW section, which consisted of two major areas: (a) Psychosocial Risk Factors (PRFs; Table A1 of Appendix A), which contains the 22 items of workload and pace of work demands (2 items), high responsibility and dangerous demands (3 items), shift and schedule demands (3 items), cognitive or attentional demands (3 items), emotional demands (3 items), physical effort and environment demands (3 items), psychological harassment at work (2 items) and stressful leadership (2 items); and (b) positive psychosocial resource factors (PPRFs; see Table A2 in Appendix A), which contains 19 items distributed across the dimensions of rewards and professional development (7 items), control over work (5 items), resources to do the job (2 items), work social support climate (3 items) and values congruence (2 items). In our previous study [17], the α and reliabilities of these sections ranged from 0.73–0.85 and from 0.70–0.87, respectively, with little distance between them.

#### Ethical Considerations

This study is part of a research project HIM/2015/017/SSA.1207; “Effects of mindfulness training on the psychological distress and quality of life of the family caregiver” that was approved by the Research, Ethics, and Biosafety Commissions of the Hospital Infantil de México Federico Gómez, National Institute of Health in Mexico City. While conducting this study, we followed the ethical rules and considerations regarding research with humans currently enforced in Mexico [34] and those outlined by the American Psychological Association [35]. All participants were informed of the objectives and scope of the research and their rights in accordance with the Declaration of Helsinki [36]. The participants who agreed to participate in the study signed an informed consent letter. Participation in this study was voluntary and did not involve payment.

### 2.3. Procedures

#### 2.3.1. Data Collection

Participants were recruited via the social networks of the principal investigator and those of two Peruvian colleagues, who were psychology graduates; the social networks used were WhatsApp and Facebook. Eligibility was based on age (a minimum of 18 years) and work activity (working at the time of data collection). The purpose of the first contact with the participants was to explain the nature and content of the study briefly in writing; subsequently, the participants received a link through the same mode of communication. The participants were also invited to forward the link to their own contacts. Individuals who were interested in participating could click on the link, which led them to a page to access the survey on the web platform. The materials were arranged the same for all participants and included a participation consent form (which provided information concerning the objective of the study, the voluntary nature of participation, the anonymity of responses, participants’ freedom to stop responding at any time, the confidential treatment of the data, and the absence of participant traceability). Participant eligibility after data collection was based on (a) contractual membership in a Peruvian labor institution in the context of the participant’s most recent job and (b) a minimum working time of six months.

#### 2.3.2. Analysis

The evaluation of internal structure and dimensionality was performed by using two approaches: nonparametric and parametric. These approaches are explained below.

#### Nonparametric Approach

Each item-theoretic group was evaluated in terms of its essential unidimensionality [37], that is, the existence of a dominant factor alongside secondary and trivial factors. This evaluation was conducted using two nonparametric item response theory approaches. The first nonparametric approach was the DETECT (Dimensionality Evaluation to Enumerate Contributing Traits index; [8]) algorithm, which applies to ordinal items (Poly-DETECT; [8,38]). This approach is based on conditional covariance between pairs of items and detects whether a group of items is essentially unidimensional or is multidimensional but configured as a simple structure. Due to the small number of items in each content group and to avoid the false identification of unidimensional or multidimensional content [38], the multidimensionality of two content items presented contiguously in the PROPSIT was tested. For example, the factors of workload and pace of work demands were included jointly in the multidimensionality assessment with high responsibility and dangerous demands; the factor of shifts or schedules demands was combined with cognitive or attentional demands; and similar combinations were employed with respect to the following content. Due to the theoretical independence of this jointly assessed content, it was expected that this set would be identified as multidimensional but with an approximately simple structure; that is, we anticipated to find that the items would function as one dimension within a multidimensional measure.

Interpretive guidelines for DETECT vary in terms of magnitude; for example, Kim [39] proposed values of <0.10 (unidimensionality), <0.5 (weak dimensionality), <1.0 (moderate dimensionality), and >1.0 (strong dimensionality); Roussos and Ozbek [40] proposed <0.20 (essential unidimensionality), >0.20 (weak to moderate multidimensionality), >0.40 (moderate to strong multidimensionality), and >1.0 (strong multidimensionality). In the present study, both criteria were applied. Alongside Poly-DETECT, the ASSI (Approximate Simple Structure Index; [41]) and the RATIO index [41] were applied as complements to detect approximately simple structures, indicating the optimal classification of items into clearly differentiated dimensions. The ASSI and RATIO ranged from 0 to 1, and suggested cutoff points were used to indicate essential unidimensionality (ASSI < 0.25, RATIO < 0.36), deviant unidimensionality (ASSI > 0.25, RATIO > 0.36), and obvious simple multidimensional structures (ASSI and RATIO > 1.0). Due to the relatively small sample size, an intervalidation test was conducted by segmenting the sample into a training and a validation sample [10]. The nonparametric detection of multidimensionality in the PROPSIT was completed with a visual structure search procedure using a hierarchical cluster analysis (the Ward method), and this element was represented visually with dendrograms. The R program *sirt* [42] was used in this context.

Since the joint use of other procedures to verify DETECT results has proven to be an effective and valid strategy [9,11], without departing from a nonparametric approach, we implemented the Automated Item Selection Procedure (AISP; [43]), a procedure that segments a set of items into subgroups with properties of the monotonic homogeneity model and thus achieves a Mokken scale to determine the score [44]. The procedure starts by looking for items with minimum H scalability coefficients of 0.30 [45] and continues up to other H levels, which are determined a priori. In an exploratory approach to search for structures or subscales, AISP helps identify a set of scalable items with a minimum satisfactory level, i.e., a scale on which the latent variable can be psychometrically isomorphic with the observable score [43,45]. The AISP results define a Mokken scale but not always the property of unidimensionality [46]; therefore, these results were contrasted and evaluated jointly with those of the Poly-DETECT algorithm. The R program *mokken* [44] was used.

#### Parametric Approach

Since there is a predefined structure for the configuration of the PROPSIT items, examination of the dimensions of the PROPSIT items was carried out by employing the semiconfirmatory factor analysis procedure [47,48], which constructs a Procrustes-type rotation for a matrix that is defined a priori (the target matrix) with respect to the relationships among the items contained in the factors of the measurement model. The target matrices for the dimensions of PRFs and favorable resources are shown in Appendix A.

The mode of estimation used for factor extraction was unweighted least squares (ULS; [48]), a method that produces approximately accurate results when there is a moderate or strong absence of normality and distributional skewness of the data when the number of factors is greater than 3 (as with the present data; [49,50]) and the indicators are ordinal [51,52]. The process for evaluating model fit employed robust estimates, and in the present study, a correction was applied to the mean of and variance in the fit χ^2^ statistic (ULSMV; [52,53]). Considering the 7 possible response options to the PROPSIT items, the ULS estimator was applied to the Pearson correlations between the items. To evaluate the degree of fit of the models to the data, the congruence coefficient (*C*; [54]) was used; this coefficient varies between −1.0 and 1.0 and quantifies the degree of numerical similarity between the result of the factor analysis and the established a priori target matrix. This coefficient indicates an acceptable (>0.85) or high similarity and practical equality (>0.95) between the factor loadings in the comparison [55]. Factor analysis was performed using the *Factor* program [56].

## 3. Results

### 3.1. Detection of Potential Response Biases

Prior to the analysis of the internal structure of the PROPSIT, by employing the Mahalanobis distance *D^2^* score method, we identified 48 participants (13.3%) as exhibiting a highly inconsistent response pattern with respect to all PROPSIT items (41 items). For the detection of highly consistent responses, using the longstring method, we found six participants (1.6%) to have provided equal sequential responses to between 22 and 41 items. The combined prevalence of participants with possible response biases was 14.9% (n = 54), including 23.4% and 8.4% of the samples of educators and general workers, respectively. These 54 participants were removed from the database prior to the analysis of the internal structure of the PROPSIT.

### 3.2. Nonparametric Assessment of Dimensionality

#### 3.2.1. Poly-DETECT Algorithm

Table 2 shows the summary coefficients of the Poly-DETECT algorithm and the complementary coefficients. It is noted that each set is a multidimensional grouping of items in the PRF area (M_poly-DETECT_ = 26.08), which was larger than the list of favorable resource items, but in aggregate, all Poly-DETECTs were substantially >1.0. The multidimensionality in each set corresponds to the dimensionality expectations stated in the study. Regarding the ASSI and RATIO, simple multidimensional structures were strongly present in Sets 3 and 4 and moderately present in Sets 1 and 2.

The distribution of psychosocial risk factor items in the subscales derived from Poly-DETECT and Mokken scaling analysis (MSA) is also shown in Table 2. In Set 2 (workload demands, shift and schedule demands and cognitive or attentional demands) and Set 3 (emotional demands and physical effort and environment demands), the items were distributed according to their theoretical structure, indicating convergence in terms of their ASSI and RATIO values (=1). Set 1 was moderately effective because Item 2 was not maintained in the context of workload and pace of work demands, and Set 4 unified all items, except Item 22, whose coding was inverted, into a single dimension.

**Figure 1:**
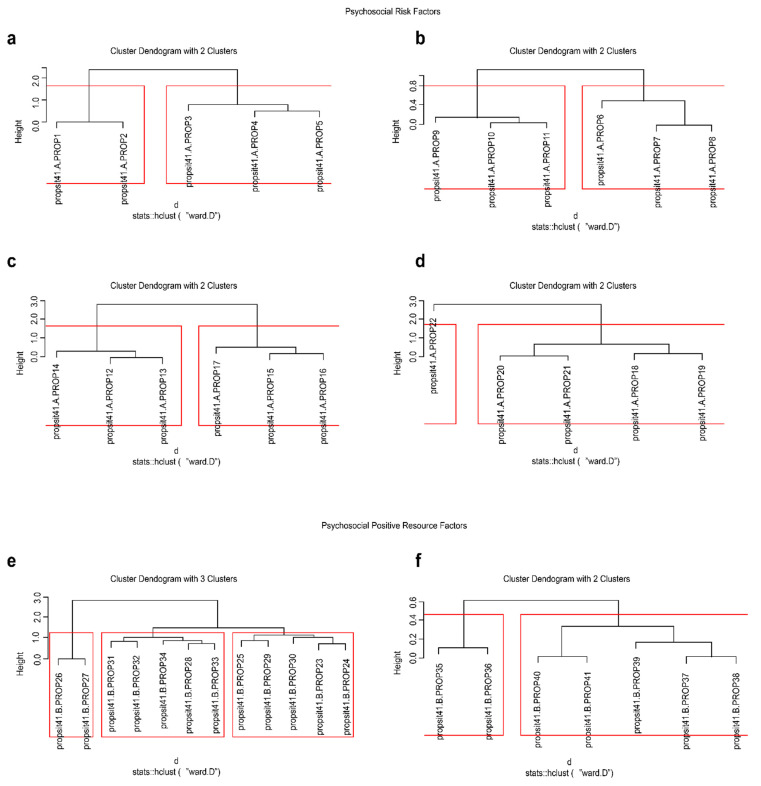
Dendrogram of the cluster analysis of PROPSIT items. Item sets in the figure: (**a**) (set 1, Table 2), (**b**) (set 2, Table 2), (**c**) (set 3, Table 2), (**d**) (set 4, Table 2), (**e**) (set 1, Table 3), and (**f**) (set 2, Table 3).

On the other hand, with respect to PPRFs, almost all items showed theoretical consistency in terms of their expected structure (Table 3). In set 1, recordable items formed a separate cluster from their expected scale, and in set 2, all items were also grouped theoretically. On the other hand, set 1 was below the cutoff point with respect to both indicators, although only slightly so in the context of ASSI; set 1 identified marginal unidimensionality in both content areas (rewards and professional development and tasks that benefit people and society), suggesting high covariation between the two. Figure 1 presents the classification dendrograms of the PRF and PPRF items.

#### 3.2.2. Automated Item Selection Procedure (AISP)

The AISP algorithm for the content regarding psychosocial risks (Table 2) was generally concordant with the results of the Poly-DETECT procedure. Specifically, set 3 was completely concordant with the theoretical model, while set 4 was concordant with the results of Juárez-García and Flores-Jiménez [17], except with respect to the item that required recoding (item 22). Except for Items 3 (workload demands) and 6 (shift and schedule demands), sets 1 and 2 were appropriately distinguished. The discrepancies found for these items were highlighted at the H level >0.50, a level of scalability that is considered high. Regarding favorable resources (Table 3), a discrepancy was observed with respect to the Poly-DETECT results because AISP was more likely to detect the merging of scales into a single scale in each set evaluated. The items that required recoding (items 26 and 27) were differentiated from the rest of the items, as was the case in the Poly-DETECT results.

### 3.3. Parametric Assessment of Dimensionality

#### 3.3.1. Psychosocial Risk Factors

Two iterations were conducted: the first iteration focused on the evaluation of the theoretical-empirical structure of the PROPSIT, and the second iteration represented the fine-tuning of the model.

#### First Iteration

Table 4 shows the results of semiconfirmatory factorization. Column C shows the congruence between the hypothesized configuration of each item and the resulting configuration. Considering this measure of fit, the configuration in the sample of educators was highly inconsistent, except for factor F7, which showed better fit; in the sample of general workers, greater consistency was found, except with factors F1, F3 and F4. In the overall sample, congruence was below the acceptability criterion (<0.85), with factors F5, F6 and F7 showing greater congruence.

The content related to workplace bullying and stressful leadership was combined into one factor, except for Item 22. Item 22 needed to be recoded to allow it to be interpreted alongside the rest of the items in its initial grouping (i.e., stressful leadership), but in the results, it shifts to other possible dimensions with higher loadings than its factor loading in its initial group, and this mobility is not constant across the samples.

The groups of items related to physical effort and environment demands were combined into a single factor, linking the physical demands placed on the worker with the physical characteristics of the work environment. The items describing emotional responses and interpersonal situations remained independent from the other items and were therefore kept distinct. The content related to cognitive or attentional demands was combined with that related to workload and pace of work demands, forming a dimension pertaining to workers’ cognitive responses to the demand for speed at work and the amount of work assigned.

The items that were conceptually elaborated to measure dangerousness and importance, as well as rotations and long hours of night work, were unstable when integrated and varied inconsistently with respect to the rest of the content. These items, alongside others, also showed a certain degree of factor complexity on at least two dimensions, indicating that such items may represent more than one dimension. For example, Item 3 (interruptions to completing tasks on time) and Item 5 (dangerous activities) exhibited such characteristics and were underlined when they were equal to or greater than 30. Other items also showed complexity, but to a lesser degree, when their loadings on other factors were approximately 0.20 (e.g., Items 18, 19, and 20). In conclusion, theoretically coherent, moderately stable clusters of items were detected in the overall sample and in the two separate subsamples, but items that exhibited factorial complexity and dimensional mobility, which were not replicable across the two samples, were also identified.

#### Second Iteration

For the subsequent iteration of the internal structure evaluation, we considered (a) the objective of the present project (to adapt the PROPSIT to a different context with some degree of generalizability for its constructs) and (b) the results obtained from the dimensionality test of the PROPSIT (the stability of the items contained in the dimensions and the degree of factorial complexity). Accordingly, a model featuring the most stable items and a lower level of factorial complexity was proposed. Items 3, 4, 5, 7, 8, 9 and 22 had previously been removed due to (a) the empirical inconsistency shown in the first iteration and (b) the fact that these items overlapped with the items that were removed in the final analysis by Juárez-García and Flores-Jiménez [17].

The factorial configuration in this final iteration is shown in Table 5, with the revised heading of Revised Target Matrix. The fit of this modified model to the total sample can be seen under the heading of Estimation and Fit in Table 5, which indicates a structure with high factor loadings on the dimensions themselves (>0.53) and predominantly low factor loadings (<0.15) on the divergent dimensions. All the congruence coefficients (C) for the items, dimensions and total factor solution were greater than 0.93. According to the content of the items, the factors retained in this final solution, resembling the factors defined in the study by Juárez-García and Flores-Jiménez [17], were psychological demands (F1), physical demands (F2), psychological harassment (F3), and emotional demands (F4). The correlations among these dimensions show a tendency toward moderate relationships with sufficient independence among them.

#### 3.3.2. Positive Psychosocial Resources

As in the results for the internal structure of the PRFs, two iterations were made pertaining to the enabling resource factors: the first iteration focused on the evaluation of the theoretical-empirical structure of the PROPSIT, and the second iteration pertained to the refinement of the model.

#### First Iteration

The results are shown in Table 6. In the overall sample, more than half of the items obtained an appropriate fit as measured by the congruency coefficients (C > 90), and factors F2 and F4 demonstrated a better fit than factors F1 and F3. The overall fit did not reach an acceptable level (C < 0.85), which suggested the need for an empirically based restructuring of the measurement model. This pattern was partially replicated in the separate samples, as in both samples, the fit was excessively poor for factors F1 (for the educator sample) and F3 (for the overall sample).

The Pearson correlation between the item C coefficients in each sample was 536, indicating a moderate resemblance between samples. The apparent source of discrepancy was found in Items 23, 24, 25 and 35, in which high factorial complexity and instability in terms of configuration were observed, as well as in Items 28 and 29, with respect to the subsamples (general workers and educators); Items 35 and 36 also showed factorial complexity and mobility toward other dimensions, especially toward the rewards and professional development dimension, as occurred in the study by Juárez-García and Flores-Jiménez [17].

#### Second Iteration

For the subsequent iteration of the internal structure evaluation, we considered (a) the objective of the present project (to adapt the PROPSIT to a different context with some degree of generalizability for its constructs) and (b) the results obtained from the dimensionality test of the PROPSIT (the stability of the items contained in the dimensions and the degree of factorial complexity). Therefore, a modified model, which appears in Table 7 under the heading Revised Target Matrix, was proposed for the PRFs. Items 26 and 27 were removed due to (a) phrasing contrary to the construct, which can be induced by method variance, and (b) their failure to show low factor loadings in the study by Juárez-García and Flores-Jiménez [17]. Items 28 and 29 were also removed due to (a) low factor loadings and factorial complexity and (b) the fact that they were also removed in the abovementioned study. All configuration coefficients at the item, factor, and total solution levels demonstrated a high magnitude (C ≥ 0.95), suggesting that the revised RF configuration optimally identified the factors. The factorial simplicity of the items was also a source of this high congruence. The content of these factors is coherent with that of the factors used in the study by Juárez-García and Flores-Jiménez [17] and can be divided into rewards and resources (F1), social support climate (F2), and control over work (F3).

It is concluded that, similar to the study by Juárez-García and Flores-Jiménez [17], the PRFs in this study include psychological demands, physical demands, psychological harassment, and the additional factor of emotional demands, which emerged independently. In terms of favorable resources, rewards and resources, a climate of social support and control over work emerged as important factors. In summary, this relocation of the items with respect to the relevant dimensions showed greater structural validity as well as a simple factorial configuration.

## 4. Discussion

### 4.1. Nonparametric Assessment

When the number of latent dimensions was evaluated, the variability of responses pertaining to the number of latent dimensions according to methods based on linear modeling was very large, and the consensus helped establish a reasonable range that moderately fit the theoretical dimensions. In accordance with the objective of the present study, the results concerning the number of dimensions differed only slightly from the number of dimensions identified in the study by Juárez-García and Flores-Jiménez [17], and the exploratory approach at this point suggested the work’s replicability. Given these exploratory results, one solution that balances empirical evidence and theoretical concerns is to draw the number of dimensions identified +/− 1 [57]. However, given the semi confirmatory framework used in this study, this option fits with the possible restructuring of the PROPSIT measurement model, as described below.

On the other hand, the nonparametric results indicated that the content of the PROPSIT is sufficiently multidimensional to be able to describe differentiated psychosocial work factors. This conclusion should be tempered to account for the methodology applied here, that is, the focus on small clusters of items greater than 1 rather than on the overall set of items. The approach to analyzing the items was necessary to reduce the effects of chance on the relatively small sample analyzed.

The differentiation of the dimensions converged moderately between the two nonparametric methods applied because DETECT and AISP-Mokken separated the specific content of each analyzed cluster, and both approaches were sensitive to the separation of negatively phrased items (Item 22 in the PRF area and Items 26 and 27 in the PPRF area). Due to this discrepancy between the two methods, the subsequent steps in defining the dimensionality of the PROPSIT in the context of the Peruvian sample required us to consider the effect of phrasing an item in a way that is opposed to the definition of the construct. At this point, it was noted that the items that required recoding to obtain a score congruent with those of the rest of the items in their dimension were not found within their expected conceptual group. These items formed an independent cluster and were isolated from the rest of their dimension. This phenomenon occurred in both the PRF (supervisor negative feedback, Item 22) and favorable resource areas (professional development possibilities, Item 26; and Job Retention Security, Item 24). These items clearly require recoding the added method variance due to the reversed phrasing used in the construct definition. Findings in this regard indicate that phrasing an item in a way that is opposed to the definition of the construct introduces variability that is not associated with the construct, and in research concerning psychosocial work factors and their effects, content that is phrased in this way may lead to a different pattern of response options [58]. This problem challenges the assumption of an equal interval measurement of the items. Response carelessness is another possible explanation for the variance introduced by these reverse-coded items [59]. Assessments of measurement equivalence also tend to identify this type of item as a source of variability in parameters when comparing groups [59]. It is likely that decisions concerning reverse-coded items will lead to their removal to avoid the introduction of irrelevant variance to the construct by the final PROPSIT model for the Peruvian sample.

### 4.2. Parametric Assessment

The objective of this element of the study was to evaluate the dimensionality of the PROPSIT items by implementing parametric methodology in the context of a different Peruvian sample. This sample consisted of regular elementary education teachers who share somewhat similar experiences of interaction with the organizational structure, types of assigned tasks, and other people involved in these educational organizations. Using a semi confirmatory factor analysis methodology, we first evaluated the expected configuration of the items contained in the dimensions, and the results were evaluated by using the coefficient of congruence and by observing the factorial complexity of the items.

Regarding the area of PRFs, inconsistent congruence was found in the results, indicating that the items did not fit satisfactorily into their expected dimensions, and some of these dimensions contained items that were theoretically unexpected. In both individual samples, the overall lack of congruence was noticeable (in each sample, the overall congruence was <0.80); however, some consistent patterns in item-factor relationships were observed between samples and in the overall sample. These patterns were evidenced by the congruence coefficients at the item level, but they could also be detected substantively because their content was theoretically explicable. Accordingly, the FRPS measurement model was modified; although such modification is not theoretically ideal, this approach is a common methodological choice in evaluating measurement models during the construction and adaptation stages. In the second iteration of the analysis, for the definition of the dimensions, the items were relocated in accordance with three criteria: the theoretical rationality of the proposed PROPSIT dimensions, the empirical results of the study by Juárez-García and Flores-Jiménez [17], and the optimization of the factors by reducing the low validity and factorial complexity of the items. As part of these decisions, it was also necessary to remove items that were unstable in terms of their dimensional location, a decision supported by the study by Juárez-García and Flores-Jiménez [17], which partially removed the same items.

The factors that were identified to exhibit high factorial congruence were psychological demands (F1), physical demands (F3), and psychological harassment (F4), that is, factors F2, F4 and F5 in the study by Juárez-García and Flores-Jiménez [17]. The factor that remained independent and that provided an additional dimension to this FRPS model was the group of items linking interactions with people and negative emotional reactions, which seems related to emotional demands (F2). In the study by Juárez-García and Flores-Jiménez [17], only Item 12 (negative emotions of other people) was included in the category of psychological demands, and the rest of the items were removed. However, this same item showed low factor loadings and moderate complexity in the exploratory analyses, which may suggest the value of modification in future studies.

With respect to some of the items, the factor complexity was clear, as expressed by moderately high factor loadings on more than one factor, but this fact does not represent a problem in absolute terms; instead, to make decisions regarding this type of structure, it is necessary to adopt a relative perspective that can make sense of this situation. For example, from an empirical angle, the factorial complexity of some items suggests that the relationships among these items and other dimensions are real, and unless these items represent random variation or systematic bias (e.g., responses with insufficient effort), the interpretation of these relationships is oriented toward a possible new way of interpreting the dimensions. For example, with respect to PPRFs, in the overall sample (found in Table 6 under the heading Total Sample), Item 28 (pleasant and rewarding work) theoretically represented the rewards and resources factor, but empirically, it was found to be associated with aspects of a job that facilitate control over performance and skill use in one’s job (i.e., the control over work factor). A nontrivial relationship was also found between this item and the factor describing social support (Table 6, the work climate and social support factor), a finding that is factorially clearer in the sample of educators. These results suggest the covariability of the positive effects of social exchanges that support the worker during his or her daily work [60]. Apparently, this relationship is conditioned by the type of position in question because this result is clearer in the sample of educators than in the sample of general workers.

Overall, our results have a range of implications. In regard to the current state of research on this new instrument, one theoretical implication is that the dimensions defined by the PROPSIT in its Mexican context may be transferable to another (specifically, Peruvian workers) Latin American context, which establishes the apparent robustness of the psychosocial risk and favorable resource factors constructed in the instrument. Second, these factors are closely aligned with factors recognized in the international literature, such as social support at work, job demands, resources, and worker control [18,19,20,21,22], and indicate that regardless of the specific content at the item level and the constructs for the study of psychosocial work factors in general, psychosocial factors should maintain a nonunified conceptualization and emphasize one but differentiated aspects of the work environment, as the conceptualization of a work environment should include PRFs and favorable resources. On the other hand, a practical implication is that the similarity of the content of dimensions used in Peru and Mexico provides a means of cross-cultural comparison and reduces measurement variability in a meta-analytic research design.

As a corollary, the results obtained support the internal structure hypotheses proposed here. Specifically, based on the results of the validation of Juárez-García and Flores-Jiménez [17] in the Mexican context, Hypothesis 1 (H1) pointed out the multidimensionality of the PROPSIT in the Peruvian sample, with associated dimensions in both. The evidence obtained from the nonparametric and parametric methodology clearly converged to expose the multidimensionality of the PROPSIT in a new (Peruvian) sample for the two types of factors, psychosocial risk and favorable resources. Therefore, this hypothesis is not rejected. The second hypothesis involved the degree of independence of the dimensions, and the results indicated that although the dimensions are related, they maintain a degree of independence that makes them distinct. Therefore, the second hypothesis is not rejected. Both nonrejected hypotheses have implications for the conceptualization of the Mexican PROPSIT adapted for the Peruvian context and even for a broader context. That is, the multidimensionality and degree of distinctiveness of the dimensions is a replicable characteristic of the general conceptualization of psychosocial work factors.

## 5. Limitations of the Study

The main potential limitation of this study was the heterogeneous distribution of one of the samples with respect to the careers and jobs of the participants, which may compromise conclusions regarding the PROPSIT’s external validity. However, the effect size of the final results (i.e., strong factor loadings, high-to-moderate reliability estimates, low factor complexity, and high congruence coefficients) may allow generalization, and taken together, these findings are nontrivial evidence for this conclusion [61,62,63]. Specifically, given that factor loadings >0.50 are considered to be high [50], the final version of the PROPSIT can achieve high internal validity and replicability for each factor. Finally, other sources of validity are needed, including the association with variables external to the measurement model and relevant to the use of the instrument, such as work stress or burnout, as well as convergent validity with other measures of psychosocial factors and test–retest reliability.

## 6. Conclusions

In terms of the need to assess psychosocial work factors, especially in contexts in which research concerning these factors is underdeveloped, the adaptation of psychosocial factor measures is a process that improves our ability to evaluate these factors within a coherent theoretical framework. One of the psychometric aspects that condition other psychometric properties is the internal structure of a measure. Considering this unprecedented initiative, the size of the sample and the importance of sensitivity analysis, this study included both a parametric and a nonparametric analysis of the internal structure of a measure. We conclude that the dimensionality of the Psychosocial Work Processes Questionnaire (PROPSIT) as applied to Peruvian workers is acceptable and converges with the dimensionality obtained in the Mexican sample referenced by the original study of this questionnaire. This similarity was expressed in the choice of most of the items for the final configuration of the instrument, the effect of phrasing items in a way that was opposed to the definition of the construct, and the interpretation of the constructs with respect to items that integrated the adapted version.

## Figures and Tables

**Table 1:** Demographic and employment characteristics of participants (before and after data cleaning).

	Full Sample (n = 359)		“Clean” Sample (n = 305)	
	n	%	n	%
Database				
General	201	56.0	184	60.3
Education	158	44.0	121	39.7
Sex				
Male	171	47.6	146	47.9
Female	187	52.1	159	52.1
Missing	1	0.3		
Place of birth				
Lima	159	44.3	147	48.2
Other than Lima	186	51.8	148	48.5
Missing	14	3.9	10	3.3
Education				
University	288	80.2	246	80.7
Technical (3 years)	47	13.1	41	13.4
Technical (<3 years)	19	5.3	18	5.9
Missing	5	1.4		
Contract				
Definitive/permanent	186	51.8	155	50.8
Seasonal/temporary	148	41.2	131	43.0
Missing	25	7.0	19	6.2
Living situation				
Alone	18	5.0	14	4.6
Alone with pet	2	0.6	2	0.7
With partner or family	315	87.7	270	88.5
With friends	3	0.8	3	1.0
Other	19	5.3	15	4.9
Missing	3	0.9	1	0.3
Classification of academic field				
Health sciences	6	1.7	5	1.6
Basic sciences	3	0.8	2	0.7
Engineering	23	6.4	21	6.9
Economic and management sciences	69	19.2	63	20.7
Humanities/legal and social sciences	193	53.8	155	50.8
Not applicable	64	17.8	59	19.3
Missing	1	0.3		
Occupational classification CIUO-08				
Directors and managers	3	0.8	1	0.3
Scientific and intellectual professionals	214	59.6	179	58.7
Technicians and mid-level professionals	55	15.3	55	18.0
Administrative support personnel	27	7.5	27	8.9
Service workers, store or market salespersons	9	2.5	10	3.3
Military, crafts, mechanical or other trade workers	28	7.8	28	9.2
Plant and machine operators and assemblers	4	1.1	4	1.3
Elementary occupation workers	1	0.3	1	0.3
Missing	18	5.0		

**Table 2:** Nonparametric assessment: psychosocial risk factor items.

	Cluster Analysis	AISP (MSA)			Poly-DETECT	ASSI	RATIO
		0.3	0.4	0.5			
Set 1					40.91	0.6	0.88
Workload and work rhythm demands							
Workload	1 ^a^	1 ^a^	1	1			
Work quickly	1	1	1	1			
Interruptions to completing tasks on time	2	2	2	0			
High responsibility demands							
Able to take important actions	2	2	2	2			
Danger	2	2	2	2			
Set 2					15.40	0.46	0.71
Shift and schedule demands							
Overtime, long hours	1	1	1	0			
Rotation/shift change	1	2	2	2			
Night work	1	2	2	2			
Cognitive or attentional demands							
Prolonged attention to tasks	2	1	1	1			
Attention to two or more tasks simultaneously	2	1	1	1			
Mental effort	2	1	1	1			
Set 3					46.41	1	1
Emotional demands							
Negative emotions of other people	1	2	2	2			
Deal with people	1	2	2	2			
Show different emotions	1	2	2	2			
Physical effort demands							
Substantial physical effort	2	1	1	1			
Uncomfortable positions	2	1	1	1			
Adverse environmental conditions	2	1	1	1			
Set 4					16.43	1	1
Psychological harassment at work							
Psychological abuse (boss, supervisors)	1	1	1	1			
Psychological abuse (peers)	1	1	1	1			
Stressful leadership							
Too much control	1	1	1	1			
Excessive rules and regulations	1	1	1	1			
Adequate feedback from supervisors (R)	2	0	0	0			

Note. AISP: automated item selection procedure. MSA: Mokken scaling analysis. Cluster analysis: classification derived from cluster analysis (the Ward method). ASSI: approximate simple structure index. ^a^ Numbers here are nominal identification for each exploratory cluster detected.

**Table 3:** Nonparametric assessment: positive psychosocial resource factor items.

	Cluster Analysis	AISP (MSA)			Poly-DETECT	ASSI	RATIO
		0.3	0.4	0.5			
Set 1					4.51	0.24	0.24
Rewards and professional development							
Fair and equitable work	1 ^a^	1 ^a^	1	1			
Motivation from salary	1	1	1	1			
Work is valued and recognized	1	1	1	1			
No professional growth opportunities (R)	2	2	2	2			
Loss of employment (R)	2	2	2	2			
Pleasant and rewarding work	1	1	1	1			
Tasks that benefit people and society	3	1	1	1			
Labor control and task content							
Free to select a job	3	1	1	1			
Use skills	3	1	1	1			
Develop skills	3	1	1	1			
Very diverse activities	3	1	1	1			
Very clear roles and tasks	3	1	1	1			
Set 2					5.28	0.52	0.65
Resources to carry out the work							
Necessary and appropriate materials	1	1	1	1			
Necessary training	1	1	1	1			
Workplace climate and social support							
Peer support	2	1	1	1			
Supervisor support	2	1	1	1			
Climate of unity/collaboration	2	1	1	1			
Congruence of values							
Value fit with organization	3	1	1	1			
Value fit with peers	3	1	1	1			

Note. AISP: automated item selection procedure. MSA: Mokken scaling analysis. Cluster analysis: classification derived from cluster analysis (the Ward method). ASSI: approximate simple structure index. ^a^ Numbers here are nominal identification for each exploratory cluster detected.

**Table 4:** Psychosocial risk factors: Semiconfirmatory factor analysis, first iteration.

	Teachers (n = 121)								General Workers (n = 184)								Total (n = 305)							
	F1	F2	F3	F4	F5	F6	F7	C	F1	F2	F3	F4	F5	F6	F7	C	F1	F2	F3	F4	F5	F6	F7	C
1. Workload	**0.31**	−0.17	0.18	**0.32**	0.07	−0.15	0.08	0.57	0.14	0.01	−0.11	**0.81**	−0.08	0.10	0.08	0.16	0.35	0.05	−0.01	**0.70**	−0.15	0.00	0.04	0.43
2. Fast-paced work	**1.03**	0.01	0.15	−0.04	0.04	−0.26	0.01	**0.96**	0.15	−0.08	−0.08	**0.78**	−0.06	0.17	0.08	0.18	**0.44**	0.09	−0.03	**0.69**	−0.15	−0.01	0.06	0.52
3. Interruptions to completing tasks on time	**0.51**	−0.17	−0.27	0.10	−0.04	**0.42**	0.04	−0.23	0.00	0.28	−0.25	−0.13	0.38	0.16	0.18	0.47	**0.71**	**0.69**	−0.13	−0.27	0.12	0.00	0.04	0.66
4. Able to take important actions	−0.01	0.32	0.20	−0.11	0.31	0.17	−0.10	0.60	−0.15	**0.76**	0.14	0.23	0.14	0.04	−0.19	**0.89**	−0.02	0.32	0.31	−0.05	0.24	0.14	−0.21	0.55
5. Danger	0.00	−0.01	**0.56**	−0.06	−0.07	**0.37**	−0.14	−0.01	0.07	**0.89**	0.07	−0.01	−0.16	0.04	0.02	**0.97**	0.09	**0.68**	0.37	0.04	−0.06	0.04	−0.11	**0.86**
6. Overtime, long hours	0.13	0.00	**0.43**	0.03	−0.22	**0.52**	−0.06	0.59	0.12	−0.08	0.15	**0.41**	0.03	0.16	0.08	0.30	0.10	0.09	0.16	**0.50**	−0.11	0.13	0.08	0.27
7. Rotation/shift change	0.14	**0.42**	0.35	0.06	0.00	−0.13	0.08	0.59	0.03	0.04	**0.67**	−0.01	0.10	−0.03	0.04	**0.98**	−0.10	0.17	**0.46**	0.14	0.06	−0.08	0.09	**0.85**
8. Night work	−0.11	−0.07	**0.80**	0.11	−0.18	−0.20	0.20	**0.90**	−0.04	0.13	**0.67**	−0.11	0.02	−0.02	0.07	**0.96**	−0.12	0.29	**0.53**	0.09	−0.09	−0.12	0.13	0.81
9. Prolonged attention to tasks	−0.11	0.00	0.26	**0.43**	0.04	0.09	−0.11	0.80	0.14	0.04	0.12	**0.76**	−0.05	0.00	−0.04	**0.96**	−0.08	−0.07	0.16	**0.78**	0.01	0.02	−0.06	**0.96**
10. Attention to two or more tasks simultaneously	0.03	0.03	−0.06	**1.04**	−0.10	0.04	−0.01	**0.99**	0.07	−0.01	0.01	**0.83**	0.07	0.10	−0.05	**0.98**	0.15	−0.03	0.09	**0.79**	0.00	0.03	−0.06	**0.97**
11. Mental effort	0.08	−0.29	0.26	0.25	**0.48**	−0.25	−0.03	0.34	0.00	0.12	−0.15	**0.82**	0.05	−0.31	0.07	**0.91**	0.05	−0.12	−0.02	**0.63**	0.23	−0.17	−0.02	**0.89**
12. Negative emotions of other people	−0.01	0.13	−0.14	0.17	**0.77**	−0.12	−0.02	**0.93**	−0.02	0.22	−0.08	0.19	**0.72**	−0.09	−0.04	**0.91**	0.02	0.05	0.02	0.17	**0.79**	−0.09	−0.07	**0.96**
13. Deal with people	−0.06	0.05	−0.13	0.08	**0.48**	0.37	0.21	0.72	0.06	−0.09	0.15	0.03	**0.71**	−0.03	0.12	**0.95**	−0.07	−0.03	0.00	0.14	**0.59**	0.08	0.23	**0.89**
14. Show different emotions	0.08	−0.05	−0.09	−0.17	**0.72**	0.19	−0.04	**0.92**	−0.06	−0.04	0.04	−0.11	**0.87**	0.06	−0.04	**0.98**	0.07	0.13	−0.07	−0.08	**0.71**	0.04	0.01	**0.96**
15. Substantial physical effort	0.01	0.04	−0.02	0.11	**0.41**	**0.35**	0.02	0.64	0.00	−0.04	−0.01	−0.09	0.08	**1.02**	−0.15	**0.98**	−0.03	−0.05	−0.08	−0.04	0.11	**0.92**	−0.08	**0.98**
16. Uncomfortable positions	−0.02	0.09	0.04	0.03	0.15	**0.71**	0.03	**0.96**	0.03	−0.02	0.04	−0.04	−0.02	**0.85**	0.03	**0.99**	0.01	0.00	−0.02	0.06	−0.07	**0.88**	0.04	**0.99**
17. Adverse environmental conditions	−0.05	−0.10	−0.02	−0.05	−0.04	**0.74**	0.06	**0.98**	0.00	0.38	−0.09	−0.11	−0.23	**0.44**	0.25	0.63	0.02	0.25	0.02	−0.19	−0.08	**0.46**	0.13	0.79
18. Psychological abuse (boss, supervisors)	0.05	0.13	0.03	−0.06	−0.11	0.03	**0.87**	**0.97**	−0.37	−0.10	0.05	0.12	0.02	0.02	**1.03**	**0.93**	0.14	−0.26	0.35	−0.24	0.01	0.00	**0.84**	**0.85**
19. Psychological abuse (peers)	−0.08	0.16	0.04	0.00	0.02	−0.03	**0.77**	**0.97**	−0.22	−0.02	0.04	0.08	−0.05	0.02	**0.92**	**0.96**	−0.01	−0.24	0.24	−0.18	0.06	0.06	**0.74**	**0.88**
20. Too much control	−0.02	−0.20	0.06	0.00	0.17	0.08	**0.69**	**0.92**	**0.62**	−0.10	0.06	−0.19	0.02	0.00	**0.57**	0.65	−0.10	0.14	−0.29	0.23	0.03	0.04	**0.82**	**0.89**
21. Excessive rules and regulations	0.07	−0.17	0.13	−0.03	0.11	−0.01	**0.68**	**0.94**	0.36	0.17	−0.05	−0.04	0.07	−0.10	**0.52**	0.77	−0.01	0.21	−0.16	0.16	0.05	−0.05	**0.69**	**0.91**
22. Feedback from supervisors (R)	−0.11	0.19	0.10	−0.10	0.35	−0.08	−0.14	0.29	0.02	−0.04	0.04	**0.57**	−0.03	0.01	−0.14	0.22	−0.10	−0.27	0.04	**0.46**	0.08	0.11	−0.13	0.22
Factor fit (C)	0.77	0.1	0.71	0.79	0.77	0.7	**0.89**	0.71	0.22	0.84	0.81	0.7	**0.92**	**0.9**	**0.86**		0.57	0.78	0.62	0.68	**0.92**	**0.93**	**0.89**	0.78

Note. Factor loading: bolded items indicate strong factor loadings on the corresponding dimension or a satisfactory level of congruence. Underlined items indicate factor loading on another dimension at ≥0.30. C: congruence coefficient; congruence coefficients that meet the criterion (C > 0.85) are shown in bold. Psychosocial risk factors. F1: Workload and pace of work demands. F2: responsibility and dangerous demands; F3: shift and schedule demands. F4: Cognitive or attentional demands. F5: Emotional demands. F6: Physical effort and environment demands. F7: Psychological harassment at work.

**Table 5:** Psychosocial risk factors: final iteration (n = 305).

	Revised Target Matrix				Estimation and Fit				
	F1	F2	F3	F4	F1	F2	F3	F4	C
1. Workload	**9**	0	0	0	**0.86**	−0.12	−0.03	0.05	**0.99**
2. Fast-paced work	**9**	0	0	0	**0.91**	−0.09	−0.03	0.06	**0.99**
6. Overtime, long hours	**9**	0	0	0	**0.55**	−0.11	0.23	0.12	**0.89**
10. Attention to two or more tasks simultaneously	**9**	0	0	0	**0.86**	−0.02	0.06	−0.08	**0.99**
11. Mental effort	**9**	0	0	0	**0.62**	0.19	−0.23	−0.05	**0.90**
12. Negative emotions of other people	0	**9**	0	0	0.11	**0.79**	−0.05	−0.07	**0.98**
13. Deal with people	0	**9**	0	0	0.08	**0.60**	0.08	0.19	**0.94**
14. Show different emotions	0	**9**	0	0	−0.11	**0.77**	0.08	−0.02	**0.98**
15. Substantial physical effort	0	0	**9**	0	0.00	0.13	**0.82**	−0.13	**0.98**
16. Uncomfortable positions	0	0	**9**	0	0.11	−0.05	**0.92**	−0.04	**0.99**
17. Adverse environmental conditions	0	0	**9**	0	−0.18	0.01	**0.54**	0.17	**0.90**
18. Psychological abuse (superiors)	0	0	0	**9**	−0.05	−0.09	−0.02	**0.93**	**0.99**
19. Psychological abuse (peers)	0	0	0	**9**	−0.09	−0.07	−0.02	**0.87**	**0.99**
20. Too much control	0	0	0	**9**	0.13	0.13	0.00	**0.62**	**0.96**
21. Excessive rules and regulations	0	0	0	**9**	0.10	0.16	−0.03	**0.61**	**0.96**
Factor fit (C)	-	-	-	-	**0.96**	**0.95**	**0.94**	**0.95**	**0.95**
Correlation									
F1	-	-	-	-	1				
F2	-	-	-	-	0.32	1			
F3	-	-	-	-	0.28	0.37	1		
F4	-	-	-	-	0.20	0.36	0.50	1	

Note. C: congruence coefficient; bolded items indicate congruence coefficients that meet the criterion (C > 0.85). Factor loading: bolded items indicate strong factor loading on the corresponding dimension. Psychosocial risk factors reviewed: F1: Psychological demands. F2: Emotional demands. F3: Physical demands. F4: Psychological harassment.

**Table 6:** Positive psychosocial resource factors: semi confirmatory factor analysis, first iteration.

	Total (n = 305)					Teachers (n = 121)					General (n = 184)				
	F1	F2	F3	F4	C	F1	F2	F3	F4	C	F1	F2	F3	F4	C
23. Fair and equitable work.	−0.16	0.11	0.17	0.41	0.33	0.21	0.04	−0.12	0.52	0.37	**0.59**	−0.26	0.40	0.16	0.75
24. Motivation from salary	−0.14	−0.01	**0.86**	−0.15	0.16	**0.59**	−0.15	0.15	−0.01	**0.94**	**0.91**	−0.10	0.37	−0.27	**0.88**
25. Work is valued and recognized	−0.10	0.19	**0.51**	0.11	0.19	**0.72**	0.13	0.02	0.06	**0.97**	**0.62**	−0.08	0.39	0.03	**0.84**
26. No professional growth opportunities (R)	**0.77**	0.19	0.09	0.01	**0.96**	−0.01	−0.05	0.26	−0.15	0.03	**−0.97**	0.07	0.71	0.03	0.80
27. Loss of employment (R)	**0.58**	−0.13	0.16	0.03	**0.93**	0.04	−0.22	0.31	−0.27	−0.08	**−1.18**	−0.11	1.16	−0.05	0.71
28. Pleasant and rewarding work	−0.14	**0.44**	−0.05	0.37	0.23	−0.03	0.21	−0.03	0.53	−0.05	0.19	0.45	0.03	0.24	0.35
29. Tasks that benefit people and society	0.02	**0.68**	−0.20	0.22	−0.03	−0.21	0.54	−0.12	0.27	−0.32	0.06	0.34	0.40	0.01	0.11
30. Free to select a job	0.02	**0.71**	0.00	0.11	**0.98**	0.07	**0.68**	0.03	−0.03	**0.99**	−0.11	**0.66**	0.12	0.22	**0.92**
31. Use skills	0.03	**1.03**	−0.01	−0.08	**0.99**	−0.10	**0.99**	0.09	−0.08	**0.98**	−0.22	**1.17**	0.04	−0.05	**0.98**
32. Develop skills	−0.03	**1.01**	−0.00	−0.11	**0.99**	0.07	**0.97**	0.01	−0.09	**0.99**	−0.04	**1.08**	−0.01	−0.09	**0.99**
33. Very diverse activities	−0.06	**0.87**	0.09	−0.15	**0.97**	0.00	**0.83**	0.05	−0.08	**0.99**	0.17	**0.87**	−0.06	−0.10	**0.97**
34. Very clear roles and tasks	0.05	**0.96**	0.07	−0.09	**0.99**	−0.05	**0.95**	0.07	−0.15	**0.98**	0.16	**0.71**	0.20	−0.11	**0.92**
35. Necessary and appropriate materials	0.04	0.17	**0.69**	0.05	**0.96**	0.20	0.29	**0.53**	0.06	**0.82**	0.19	0.35	0.09	0.31	0.18
36. Necessary training	−0.06	−0.01	**0.64**	0.17	**0.95**	0.02	0.14	**0.62**	0.28	**0.89**	**0.62**	−0.12	0.07	0.30	0.10
37. Peer support	0.06	−0.17	0.16	**0.81**	**0.95**	−0.27	−0.19	0.21	**0.82**	**0.89**	−0.05	−0.10	0.15	**0.90**	**0.97**
38. Supervisor support	0.01	−0.17	−0.03	**1.00**	**0.98**	−0.16	−0.13	0.17	**0.86**	**0.95**	−0.12	−0.16	0.03	**1.11**	**0.98**
39. Climate of unity/collaboration	−0.03	−0.00	−0.00	**0.85**	**0.99**	0.10	−0.12	−0.06	**0.80**	**0.97**	−0.07	0.05	−0.01	**0.95**	**0.99**
40. Value fit with organization	−0.01	0.07	0.01	**0.79**	**0.99**	0.23	0.01	−0.09	**0.64**	**0.93**	−0.09	0.18	−0.02	**0.88**	**0.97**
41. Value fit with peers	−0.02	−0.10	0.06	**0.82**	**0.98**	0.17	−0.05	−0.01	**0.61**	**0.96**	0.11	−0.16	−0.04	**0.95**	**0.97**
Factor Fit (C)	0.70	**0.90**	0.65	**0.93**	0.78	0.42	**0.92**	0.81	**0.86**	0.74	0.8	0.9	0.07	0.95	0.77

Note. Factor loading: bolded items indicate strong factor loadings in the corresponding dimension or a satisfactory level of congruence. Underlined items indicate factor loading on another dimension at ≥0.30. C: congruence coefficient; congruence coefficients that meet the criterion (C > 0.85) are shown in bold. Positive psychosocial resource factors. F1: Rewards and professional development. F2: Control over work and task content. F3: Resources to perform the job. F4: Work climate and social support.

**Table 7:** Positive psychosocial resource factors: final iteration (n = 305).

	Revised Target Matrix			Estimation and Fit			
	F1	F2	F3	F1	F2	F3	C
24. Motivation from salary	**9**	0	0	**1.08**	−0.10	−0.30	**0.95**
25. Work is valued and recognized	**9**	0	0	**0.66**	0.15	0.00	**0.97**
30. Free to select a job	0	**9**	0	−0.01	**0.67**	0.15	**0.97**
31. Use skills	0	**9**	0	−0.08	**0.99**	0.02	**0.99**
32. Develop skills	0	**9**	0	−0.04	**1.00**	−0.02	**0.99**
33. Very diverse activities	0	**9**	0	0.11	**0.83**	−0.09	**0.98**
34. Very clear roles and tasks	0	**9**	0	0.06	**0.86**	−0.02	**0.99**
35. Necessary and appropriate materials	**9**	0	0	**0.82**	0.03	−0.02	**0.99**
36. Necessary training	**9**	0	0	**0.86**	−0.12	0.04	**0.98**
37. Peer support	0	0	**9**	0.16	−0.15	**0.75**	**0.95**
38. Supervisor support	0	0	**9**	−0.06	−0.07	**0.94**	**0.99**
39. Climate of unity/collaboration	0	0	**9**	−0.02	0.09	**0.79**	**0.99**
40. Value fit with organization	0	0	**9**	−0.05	0.16	**0.80**	**0.97**
41. Value fit with peers	0	0	**9**	0.01	−0.01	**0.81**	**1.00**
Factor fit (C)				**0.97**	**0.97**	**0.97**	**0.97**
Correlation							
F1	-	-	-	1			
F2	-	-	-	0.69	1		
F3	-	-	-	0.80	0.68	1	

Note. C: congruence coefficient; bolded items indicate congruence coefficients that meet the criterion (C > 0.85). Factor loading: bolded items indicate strong factor loading on the corresponding dimension. Positive psychosocial resource factors: F1: Rewards and resources. F2: Control over work. F3: Climate of social support.

## Data Availability

The raw data supporting the conclusions of this article will be made available by the authors without undue reservation.

## Appendix A

**Table A1:** Psychosocial risk factors: target matrix for the first iteration.

	F1	F2	F3	F4	F5	F6	F7
1. Workload	**9**	0	0	0	0	0	0
2. Fast-paced work	**9**	0	0	0	0	0	0
3. Interruptions to completing tasks on time	0	**9**	0	0	0	0	0
4. Able to take important actions	0	**9**	0	0	0	0	0
5. Danger	0	**9**	0	0	0	0	0
6. Overtime, long hours	0	0	**9**	0	0	0	0
7. Rotation/shift change	0	0	**9**	0	0	0	0
8. Night work	0	0	**9**	0	0	0	0
9. Prolonged attention to tasks	0	0	0	**9**	0	0	0
10. Attention to two or more tasks simultaneously	0	0	0	**9**	0	0	0
11. Mental effort	0	0	0	**9**	0	0	0
12. Negative emotions of other people	0	0	0	0	**9**	0	0
13. Deal with people	0	0	0	0	**9**	0	0
14. Show different emotions	0	0	0	0	**9**	0	0
15. Substantial physical effort	0	0	0	0	0	**9**	0
16. Uncomfortable positions	0	0	0	0	0	**9**	0
17. Adverse environmental conditions	0	0	0	0	0	**9**	0
18. Psychological abuse (boss, supervisors)	0	0	0	0	0	0	**9**
19. Psychological abuse (peers)	0	0	0	0	0	0	**9**
20. Too much control	0	0	0	0	0	0	**9**
21. Excessive rules and regulations	0	0	0	0	0	0	**9**
22. Feedback from supervisors (R)	0	0	0	0	0	0	**−9**

Note. 9: Code indicating the location of the item, in which the factorial loading will be estimated freely. 0: value to which the factorial loading will be approximated during the Procrustes rotation. R: item to be recoded for interpretation. F1: Workload and pace of work demands. F2: High responsibility and dangerous demands; F3: Shift and schedule demands. F4: Cognitive or attentional demands. F5: Emotional demands. F6: Physical effort and environment demands. F7: Psychological harassment at work and stressful leadership.

**Table A2:** Positive psychosocial resource factors: target matrix for the first iteration.

	F1	F2	F3	F4
23. Fair and equitable work	**9**	0	0	0
24. Motivation from salary	**9**	0	0	0
25. Work is valued and recognized	**9**	0	0	0
26. No professional growth opportunities (R)	**−9**	0	0	0
27. Loss of employment (R)	**−9**	0	0	0
28. Pleasant and rewarding work	**9**	0	0	0
29. Tasks that benefit people and society	**9**	0	0	0
30. Free to select a job	0	**9**	0	0
31. Use skills	0	**9**	0	0
32. Develop skills	0	**9**	0	0
33. Very diverse activities	0	**9**	0	0
34. Very clear roles and tasks	0	**9**	0	0
35. Necessary and appropriate materials	0	0	**9**	0
36. Necessary training	0	0	**9**	0
37. Peer support	0	0	0	**9**
38. Supervisor support	0	0	0	**9**
39. Climate of unity/collaboration	0	0	0	**9**
40. Value fit with organization	0	0	0	**9**
41. Value fit with peers	0	0	0	**9**

Note. 9: Code indicating the location of the item, in which the factorial loading will be estimated freely. 0: value to which the factorial loading will be approximated during the Procrustes rotation. R: item to be recoded for interpretation. F1: Rewards and professional development. F2: Control over work and task content. F3: Resources to perform the job. F4: Work climate and social support.
